# Combination of treatments with transoral endoscopic thyroidectomy vestibular approach (TOETVA) for Graves’ disease

**DOI:** 10.1038/s41598-023-29885-2

**Published:** 2023-02-16

**Authors:** Yi-Ju Wu, Yen-Hsiang Chang, Chiajen Tsai, Yi-Chia Chan, Shun-Yu Chi, Fong-Fu Chou, Wei-Che Lin, Yi Ting Yang

**Affiliations:** 1 Department of Surgery, Jen-Ai Hospital, Taichung and Chang Gung University College of Medicine, Taichung, Taiwan 412,; 2 Department of Nuclear Medicine, Kaohsiung Chang Gung Memorial Hospital and Chang Gung University College of Medicine, Kaohsiung, Taiwan; 3 Department of Internal Medicine, Kaohsiung Chang Gung Memorial Hospital and Chang Gung University College of Medicine, Kaohsiung, Taiwan; 4 Department of Radiology, Kaohsiung Chang Gung Memorial Hospital and Chang Gung University College of Medicine, Kaohsiung, Taiwan; 5 Department of Surgery, Kaohsiung Chang Gung Memorial Hospital and Chang Gung University College of Medicine, Kaohsiung, Taiwan

**Keywords:** Endocrinology, Medical research, Signs and symptoms

## Abstract

Although the success rates of non-surgical treatments for Graves’ disease such as antithyroid medication and radioiodine ablation were good, there were still failure of treatments or intolerance for some patients. Traditional thyroid surgery could treat these patients but result in unaesthetic neck scars. Herein, we report the preliminary results of our combination of treatments with the transoral endoscopic thyroidectomy vestibular approach for Graves’ disease. A retrospective review of patients who underwent the transoral endoscopic thyroidectomy vestibular approach for the treatment of different sizes of goiters between January 2019 and December 2020 was performed. The demographic and clinical data of patients were collected. All patients were followed up for > 12 months. Each patient’s goiter size was determined using four grades—from 0 to 3. In total, 14 female patients receiving the combination treatment with > 1 year of follow-up and a median (range) age of 35 (20–48) years at surgery were included. There were two, three, four, and five patients with grade 0, 1, 2, and 3 goiters, respectively. The median (range) intraoperative blood loss was higher in grade 3 patients (100 [20–850] mL) than in grade 2 patients (20 [10–200] mL) and grade 1 and 0 patients (both < 10 mL) (*p* = 0.033). All patients had normal-looking necks with a euthyroid or hypothyroid status within 1 year. There were no complications, including re-operation for bleeding, hypoparathyroidism, vocal cord palsy, or infections. The designed combination treatment with the transoral endoscopic thyroidectomy vestibular approach for Graves’ disease provides optimal cosmetic results with a high success rate.

## Introduction

Graves’ disease (GD) is a common autoimmune disorder in which antibodies bind to the thyrotropin receptor (TRAb), resulting in hyperthyroidism^1^. Treatment options include antithyroid drugs (ATDs), radioactive iodine therapy (RAI), and surgery. Although most physicians recommend ATDs as the first line of treatment, they demonstrate some negative effects, including rashes, arthralgias, urticaria, and gastrointestinal symptoms, and major adverse events, including hepatitis or vasculitis^2^. Additionally, a high ATD failure rate of 48% was reported in a study of 720 patients^3^.

In Europe and Asia, RAI or surgery are considered a second-line treatment in cases of recurrence, whereas in the United States ATDs are used as an adjunct before RAI or surgery^4–6^. Thyroidectomy has been regarded as a prompt and assured therapy for GD; however, it has some drawbacks such as unaesthetic operative scars, the need for hospitalization, and complications related to surgery, including vocal cord paralysis, hypoparathyroidism, and excessive intraoperative bleeding^6,7^. Especially in patients with large goiters, the thyroid gland tends to show hypervascularity and achieving hemostasis is often difficult^7^. Moreover, post-thyroidectomy scars on the neck add a psychological burden on patients and affect their quality of life^8^. Therefore, clinicians should attempt to minimize the effects of scars such that no scars are visible and fewer surgical complications arise.

RAI is another effective means of treating hyperthyroidism, with minor complications and no visible neck scars^9^. However, RAI’s 1-year success rate for a grade 3 goiter is only 55% in our center^5^, while the rates reported by other studies range from 37.4 to 74%^9,10^. Until now, no methods with satisfying results and success rates for treating GD have been reported.

In this study, we retrospectively reviewed our combination treatment of transoral endoscopic thyroidectomy vestibular approach (TOETVA) with selective embolization of the thyroid artery (SETA), ATDs, RAI, and radiofrequency ablation (RFA) for treating different goiter sizes in patients with GD.

## Material and methods

For our research involving human participants including the use of images, informed consent from all subjects and /or their legal guardians were obtained for both study participation and publication of identifying images in an online open-access publication The clinical research was also approved by the Institutional Review Board of our hospital (No 202101890B0) and was conducted in accordance with the Declaration of Helsinki. We performed a retrospective review of patients who underwent thyroidectomy using TOETVA at our endocrine surgical unit to treat different goiter sizes in GD between January 2019 and December 2020. The demographic and clinical data were collected. All patients were followed up for > 12 months. The goiter size was identified by palpation and classified into four grades: grade 0, no palpable goiter; grade 1, a palpable goiter not reaching the medial edge of the sternocleidomastoid muscle; grade 2, a palpable goiter reaching the sternocleidomastoid muscle but not exceeding the lateral edge; and grade 3, a palpable goiter exceeding the lateral edge of the sternocleidomastoid muscle^5^.

The indications for choosing surgery were recorded. The exclusion criteria were any degree of Graves’ ophthalmopathy, because RAI may worsen ophthalmopathy; women planning a pregnancy or breastfeeding within 6 months of surgery; a toxic multinodular goiter or any nodules with suspected malignancy; and neck surgery or radiation before TOETVA. If a patient had no clear, absolute or relative, contraindication for surgery but expressed a preference for surgery, it was recorded as the patient’s preference.

### Preoperative management

In preparation for surgery, all patients were prescribed ATDs to achieve a euthyroid status before surgery. Beta-blockers, including propranolol, were prescribed for symptomatic or heartbeat control. Lugol’s iodine solution, using 8 mg iodide/iodine per drop, 5–7 drops daily, was administered orally for 7–10 days before the operation date.

### Operation

The TOETVA procedure, including total thyroidectomy (TT), near-total (NT), subtotal-bilateral (SB-BL), or subtotal-Downhill (SB-D) surgeries, was documented. The intraoperative blood loss (IOBL) was measured by suction bottle or gauze weight. All the specimens were fragmented inside the plastic bags, and then removal from oral incisions. Whether TOETVA was combined with intraoperative RFA, preoperative SETA, or postoperative RAI, the postoperative placement of drainage tubes and hospital stays were recorded. Drainage tube removal occurred when there was less than 30 mL/day of drainage.

### Perioperative care

Serum calcium and phosphate levels were checked every 12 h until stabilization. Patients who could discontinue calcium plus vitamin D analog supplements in the presence of normocalcemia (normal, 8.0–10.0 mmol/L) within 6 months after surgery were considered to have temporary hypoparathyroidism, whereas those who continued for > 6 months, along with below-normal serum parathyroid hormone levels (normal, 11–80 pg/mL) were considered to have permanent hypoparathyroidism. An oral thyroxine supplement was prescribed on the day of discharge for hormonal replacement, depending on the patient’s age and body weight if TT or NT was performed. Antibiotics were routinely prescribed, as previously published^11^.

### Follow-up

The thyroid functional status and goiter size were measured periodically, usually at 1–2 months after surgery, then every 3–6 months. Failure of treatment was defined as a recurrence of hyperthyroidism at 12 months, requiring either long-term ATD therapy, a repeat dose of RAI, or thyroidectomy. Conversely, successful treatment was defined as a euthyroid or hypothyroid status with a normal-looking neck at 12 months. When the patients presented with hypothyroidism under thyroxine replacement, they received long-term follow-up at least every 3 months. Other complications were also recorded.

### Statistical analysis

Numerical variables are expressed as mean ± standard deviation or median (range). A one-way analysis of variance *F*-test along with a post-hoc Tukey test for multiple comparisons was used to specify potential differences. Additionally, the nonparametric Kruskal–Wallis test was used to verify the analysis of variance *F*-test results. Categorical data in the form of rates or proportions were compared using Fisher’s exact or chi-square tests. Differences were considered significant if the one-tailed *p*-value was < 0.05. Statistical analysis was performed using SPSS software v.20 (SPSS, Chicago, IL).

### Ethics approval

The study was in accordance with the Declaration of Helsinki (as revised in 2013) and approved by the Institutional Review Board of Kaohsiung Chang Gung Memorial Hospital (No. 202101890B0).

### Consent to participate

Verbal informed consent was obtained from all patients included in the study.

## Results

In total, 14 female patients, who were non-smokers with a median (range) age of 35 (20–48) years, underwent surgery by the same surgical team. According to the clinical examination of goiter size, there were two patients with grade 0, three with grade 1, four with grade 2, and five with grade 3 goiters. The indications for surgery were intolerance to ATDs, RAI treatment failure, a preference for surgical removal, a huge mass with compression, and cosmetic appearance. The patients’ demographic and clinical data are presented in Table 1.

**Table 1:** Demographics, goiter size, and indications of the patients.

Characteristic	Value
Age, median (range)	
Years old	35 (20–48)
Sex	
Female, non-smoker	14
Goiter size	
Grade 0	2
Grade 1	3
Grade 2	4
Grade 3	5
Indication	
Intolerance to ATDs	4
Failure of I-131	1
Preference for removal	3
Huge mass with compression	7
Cosmetic appearance	11

Data are presented as the number of patients unless otherwise noted.

*ATD* anti-thyroid drug.

Table 2 shows the operative variables for the different groups according to goiter size (grade 0–3). No significant differences in operative time were noted among the four groups (*p* = 0.779). The median (range) IOBL was higher in grade 3 patients (100 [20–850] mL) than in grade 2 patients (20 [10–200] mL) and grade 1 and 0 patients (both < 10 mL) (*p* = 0.033). The methods of thyroid resection performed included: TT/NT in one, three, and one patient(s) with grade 0, 1, and 2 goiters, respectively; ST-D in two patients, one grade 0 and one grade 2 goiters; and ST-BL in seven patients, two grade 2 and five grade 3 goiters. The thyroid gland weight after resection was not significantly different among the four groups (*p* = 0.101). Intraoperative RFA was performed in one patient with a grade 2 goiter and in four patients with grade 3 goiters. Preoperative SETA was utilized in one patient with a grade 2 goiter and in one with a grade 3 goiter. Postoperative RAI was performed with a fixed dose of 7 mCi. Two patients with grade 2 and two with grade 3 goiters received one dose of RAI, whereas three patients with grade 3 goiters received a second dose of RAI 6 months later. Postoperative drainage tubes were placed in only two patients with grade 3 goiters. The median (range) postoperative hospital stay was slightly longer in patients with grade 3 goiters (5 [3–7] days) than in patients with grade 2 (4 [3–4] days), grade 1 (3 [3–3] days), and grade 0 (2.5 [2–3] days) goiters (*p* = 0.022).

**Table 2:** Operative variables according to goiter size grade.

Operative variables	Grade 0 (n = 2)	Grade 1 (n = 3)	Grade 2 (n = 4)	Grade 3 (n = 5)	Total N	*p* value
Operative time (min)						
Mean ± SD	187.5 ± 10.6	200 ± 13.2	196.3 ± 11.1	213.8 ± 49.4	–	0.779
IOBL (mL)						
Median (range)	< 10	< 10	20 (~ 10–200)	100 (~ 20–850)	–	0.033
Resection						
TT/NT	1	3	1	0		–
ST-D	1	0	1	0		–
ST-BL	0	0	2	5		
Weight of resection (g)						
Mean ± SD	19 ± 1.4	52.6 ± 19.0	41.0 ± 12.7	52.4 ± 9.9	–	0.101
RFA						
No	2	3	3	1	9	
Yes	0	0	1	4	5	
SETA						
No	0	0	0	0	0	
Yes	0	0	1	1	2	
Radioiodine I-131						
Null	2	3	2	0	7	
One dose (> 2 weeks)	0	0	2	2	4	
2nd dose (> 6 months)	0	0	0	3	3	
Drainage tube placement						
No	2	3	4	3	12	–
Yes	0	0	0	2	2	–
Postoperative hospital stay (days)						
Median (range)	2.5 (2–3)	3 (3–3)	4 (3–4)	5 (3–7)	–	0.022

Data are presented as number of patients, unless otherwise noted.

*SD* standard deviation, *IOBL* intraoperative blood loss, *TT* total thyroidectomy, *NT* near-total thyroidectomy, *ST-D* subtotal-Downhill, *ST-BL* subtotal bilateral, *RFA* radiofrequency ablation, *SETA* selective embolization of thyroid artery.

**Figure 1:**
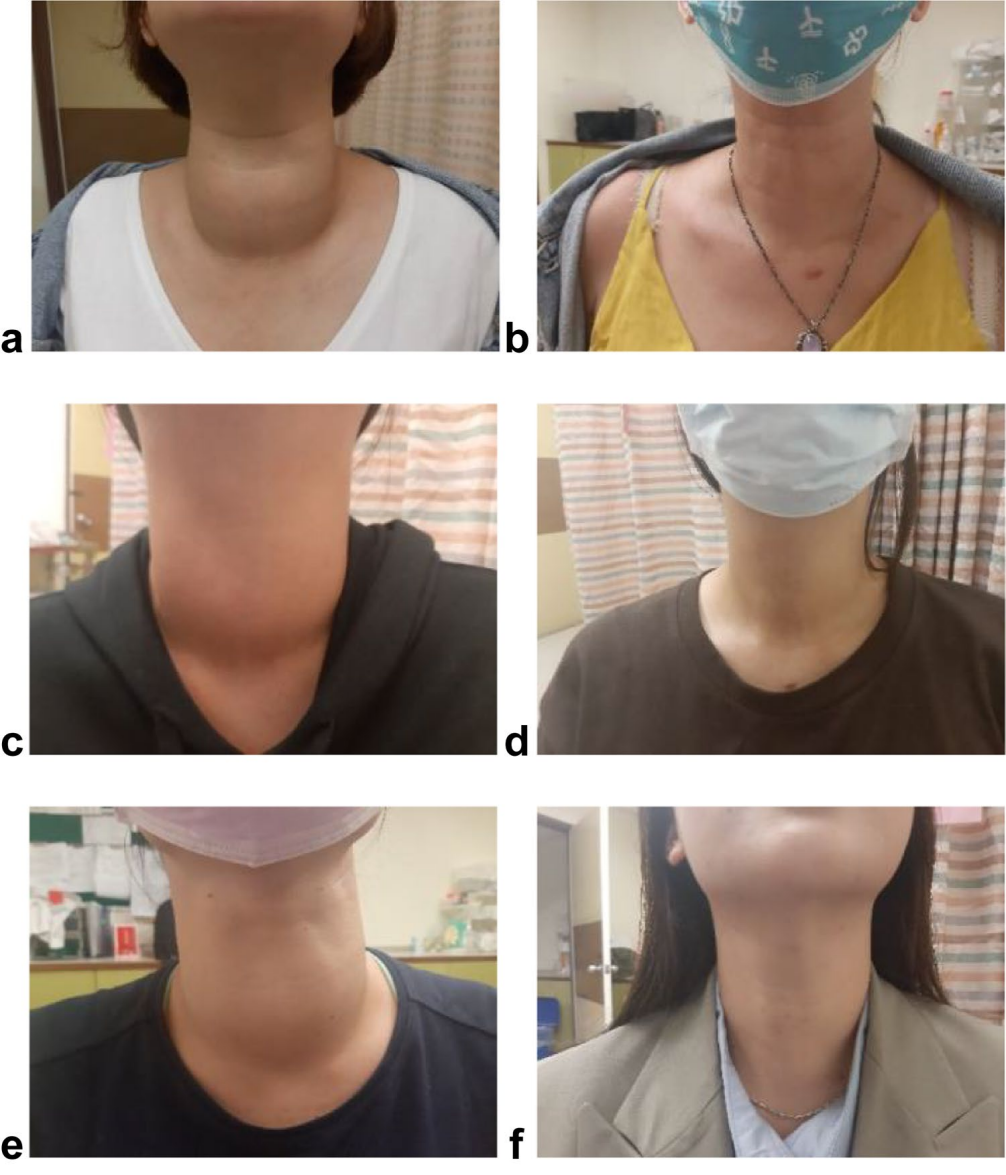
Photographs of large goiters. (**a**,**c**,**e**) Preoperative; (**b**,**d**,**f**) Postoperative.

There were no reports of complications, including re-operation for bleeding, temporary or permanent hypoparathyroidism, either unilateral or bilateral vocal cord palsy, or infections (Table 3). The 3 patients with grade 3 goiters receiving 2nd dose of RAI returned into hypothyroidism after 3 months. At 12 months after surgery, 1 patient was in a euthyroid state and 13 demonstrated hypothyroidism. However, one patient with grade 2 goiters was diagnosed with recurrent hyperthyroidism at 18 months of follow-up; a second RAI dose of 7 mCi was administered, and the hypothyroidism was evaluated after 3 months. In sum, the treatment failure or recurrence as 28.6% were seen in 4 patients receiving 2 doses of RAI in this follow-up. They all succeeded in getting hypothyroidism after that. Pre- and postoperative photographs of large goiters are shown in Fig. 1.

**Table 3:** Postoperative complications and thyroid status.

Postoperative complications and thyroid status	Grade 0	Grade 1	Grade 2	Grade 3	Total
Complications					
Reoperation for bleeding	0	0	0	0	0
Hypoparathyroidism	0	0	0	0	0
Unilateral vocal cord palsy	0	0	0	0	0
Bilateral vocal cord palsy	0	0	0	0	0
Infection at 12 months	0	0	0	0	0
Thyroid status					
Hyperthyroidism	0	0	0	0	0
Euthyroid	0	0	1	0	1
Hypothyroidism	2	3	3	5	13

Data are presented as number of patients.

## Discussion

The optimal GD treatment is controversial, despite the American Thyroid Association guidelines published in 2016^12^. Oral administration of ATDs is still the first treatment choice for a newly diagnosed GD patient, although it is associated with high failure and relapse rates using a single 18-month ATD course^3^. After failure, ATD continuation is not advocated because it usually requires life-long therapy and carries the risk of adverse effects such as agranulocytosis and hepatotoxicity^13^.

An alternative, non-surgical treatment is RAI. The fixed-dose RAI of 7 mCi for GD at our center demonstrated a success rate of 70.9%, and the mean period to achieve success was approximately 3 years, with a low first-year success rate of 37.1%^5^. Patients with grade 3 goiters exhibited a lower success rate (55.1%) than those with grade 0–2 goiters^5^. The sole independent factor determining the outcome is the goiter size^5^. Furthermore, using a higher RAI dose of > 15 mCi was unsuccessful in treating patients with large goiters^14^. Additionally, using an RAI dose of > 10 mCi increases the mortality of solid cancer patients by 1.08 times^15^. Therefore, decreasing the goiter size during surgery to at least grade 2 and using the safer and more effective 7-mCi dosage of postoperative RAI are crucial.

In contrast to the two non-surgical treatments, surgery is another option^16^. However, because the general population tends to fear surgery, as it can result in complications and unpleasant scars, it is underused. Surgical complications include transient or permanent hypoparathyroidism, hematomas requiring reoperation, and unilateral or bilateral recurrent laryngeal nerve injuries^16^. Moreover, the neck scars observed after thyroidectomy significantly affect the patient’s psychological well-being and quality of life^8^.

The novel TOETVA technique is safe and feasible to avoid a cervical scar^17,18^. Additionally, the laparoscope provided good resolution and magnification, so the parathyroid glands were well preserved in the TT/NT/SB-D surgeries and no parathyroid glands found in these specimens*.* The postoperative complications and cosmetic results are also reliable in well-selected patients wishing to avoid a cervical scar^19,20^. Thus, we designed this combination of treatments to achieve optimal cosmetic results. We used TOETVA to avoid neck scars, combined with intraoperative RFA to achieve hemostasis and volume reduction; preoperative SETA to decrease blood flow to the thyroid gland and IOBL^21^; and postoperative RAI, to ablate the residual gland.

The success rate of SETA for GD ranges from 63 to 78%, which is similar to RAI’s^21,22^. However, it carries the possibility of severe complications, including infection or thyroid storm, and should be considered only as an alternative in patients who cannot or do not want to undergo surgery or RAI^21–24^. In our study, preoperative SETA was performed 3 days before surgery. Only three vessels (the bilateral superior and left and right inferior arteries) were embolized, not to treat GD or correct hyperthyroidism, but because we assumed that sparing either side of the inferior artery would maintain the parathyroid function and prevent thyroid gland necrosis. Additionally, SETA performed on the three vessels was expected to decrease the thyroid gland’s blood flow and reduce IOBL, such that a large amount of thyroid hormones would not be released, causing thyroid storm. Antibiotics were also prescribed for the short term to avoid infection during the perioperative care after SETA. We resected most of the thyroid gland less than 3 days following embolization.

Thermal ablation using RFA under echo guidance is a new, minimally invasive alternative to surgery in patients with benign thyroid nodules, recurrent thyroid cancer, or autonomously functioning nodules^25,26^. There were no definitive reports for treating GD with RFA ablation. However, RFA-assisted liver resection with reduced blood loss and improved hemostasis was reported^27,28^. Hence, we adopted RFA for intraoperative hemostasis and volume reduction for enlarged thyroid glands. The aim of estimated residual glands after RFA was to achieve at least less than a grade 2 goiter, because at our center the success rate of RAI with 7 mCi for a grade 2 goiter was 70%. Usually, we remove and ablate the thyroid glands as much as possible during surgery. If difficulty in mobilizing the thyroid gland presents, or severe adhesion due to thyroiditis is observed, isthmectomy and bilateral partial gland removal are performed and the residual glands are ablated with RFA as much as possible. The key safety point for RFA is to maintain a safe distance from the thyroid bed using ultrasonography to avoid injuries to the recurrent laryngeal nerve, parathyroid glands, and trachea.

As previously mentioned, the success rate of RAI for a large goiter is not satisfactory. Therefore, we designed this combination of treatments using RAI as an adjuvant after surgery. In this study, the treatment failure or recurrence were seen in 4 patients including 1 with grade 2 and 3 with grade 3 goiters. The cause of the treatment failure or recurrence could be due to inadequately reducing the size of thyroid glands by TOETVA during surgery. So they returned to hyperthyroidism soon. Although the treatment failure or recurrence occurred, the 2^nd^ dose of RAI still effectively salvaged these patients. Postoperative RAI with a low dose of 7 mCi could be an effective and safe procedure after reducing the gland size following the combination treatment with TOETVA.

### Limitations

Our study has some limitations. First, this is a retrospective study, and we reviewed all possible methods for treating GD to formulate a new treatment approach. However, there were no previous reports to use for guidance. Nevertheless, our study is unique and demonstrated an acceptable success rate and optimal cosmetic results within 1 year of follow-up. Second, we didn’t perform routine laryngoscopy to access vocal cord function. So the results of complication may be some bias or subjective. Third, we had a small sample size and limited experience. Therefore, we did not have enough data to clarify the different strategies of combination treatments for different goiter sizes. Fourth, it has been reported that the TRAb assay can predict the recurrence rate of GD at a level of > 10 IU/L, and remission at a level of < 4.5 IU/L^29^. However, we did not collect the TRAb data regularly, and the follow-up period was not sufficient to report the recurrence or remission rates. Currently, one of our patients with a grade 2 goiter and the longest follow-up has a TRAb level of 6 IU/L at 2 years.

## Conclusion

Although the transverse cervical incision for treating GD is a rapid treatment modality, it requires experienced surgeons to avoid complications and results in long, unaesthetic neck scars that greatly impact the patients’ psychological well-being. Hence, our TOETVA method for avoiding neck scars, in combination with other hemostasis procedures before and during the operation for bleeding control, is practical. A low dose of RAI as a rescuing method following surgery is an effective and safe option to avoid increasing the mortality risk of solid cancers. Thus, this combination treatment provides optimal cosmetic results. Our research needs a larger sample size to clarify the different strategies to adopt for different goiter sizes, and longer follow-up periods to evaluate the relapse or recurrent cases in the future (Supplementary Information S1).

## Supplementary Information

Supplementary Information.

## Data Availability

All data generated or analysed during this study are included in the published article and its supplementary information file.
