# Supplementary material for: Combination of treatments with transoral endoscopic thyroidectomy vestibular approach (TOETVA) for Graves’ disease
Source: Sci Rep. 2023 Feb 16;13:2764. doi: 10.1038/s41598-023-29885-2 (PMC9935526; doi:10.1038/s41598-023-29885-2)
Supplement: Supplementary file 1 — Supplementary Information. [file 41598_2023_29885_MOESM1_ESM.docx]

| Number | Age | Grades | indications | SETA | Weight ( gm) | RFA | Resetcion | I-PTH | Operative time | Blood loss | free T4 | T3 | TSH | Drainage | hospital stay | Radioiodine I-131 frequency |
| --- | --- | --- | --- | --- | --- | --- | --- | --- | --- | --- | --- | --- | --- | --- | --- | --- |
| 1 | 38 | 3 | 3 | 0 | 32.7 | 1 | 4 | 25 | 360 | 150 | 0.4 | 61.7 | 8.387 | 1 | 5 | 2 |
| 2 | 20 | 3 | 3 | 0 | 30 | 1 | 4 | 25 | 400 | 650 | 0.6 | 58 | 2.785 | 1 | 5 | 2 |
| 3 | 21 | 2 | 3 | 0 | 22.8 | 1 | 4 | 20 | 270 | <10 | 0.48 | 100 | 0.014 | 0 | 3 | 0 |
| 4 | 35 | 3 | 4 | 0 | 27.4 | 1 | 3 | 22 | 235 | <10 | 1.45 | 210 | <0.01 | 0 | 2 | 0 |
| 5 | 23 | 1 | 2 | 0 | 21.4 | 0 | 2 | 26 | 240 | <10 | 2.35 | 294 | <0.01 | 0 | 2 | 0 |
| 6 | 29 | 2 | 3 | 0 | 64 | 0 | 3 | 29 | 250 | <10 | 0.4 | 66 | <0.01 | 0 | 2 | 0 |
| 7 | 24 | 0 | 4 | 0 | 14 | 0 | 1 | 40.1 | 300 | <10 | 0.98 | 60 | 0.013 | 0 | 2 | 0 |
| 8 | 29 | 2 | 3 | 0 | 24 | 1 | 4 | 30 | 360 | 80 | 1 | 87 | 1.24 | 0 | 2 | 1 |
| 9 | 29 | 3 | 2 | 0 | 60 | 0 | 1 | 26.3 | 365 | <10 | 0.94 | 117.5 | <0.01 | 0 | 2 | 0 |
| 10 | 30 | 2 | 3 | 0 | 64 | 0 | 4 | 60 | 290 | <10 | 0.55 | 101 | 0.013 | 0 | 2 | 2 |
| 11 | 45 | 3 | 3 | 1 | 20 | 0 | 4 | 37 | 270 | <10 | 0.59 | 74.2 | 0.015 | 0 | 2 | 2 |
| 12 | 38 | 1 | 4 | 0 | 36 | 0 | 1 | 34 | 240 | <10 | 1.64 | 110 | <0.01 | 0 | 2 | 0 |
| 13 | 41 | 1 | 2 | 0 | 70 | 0 | 4 | 25 | 245 | <10 | 1.47 | 129.6 | <0.01 | 0 | 3 | 0 |
| 14 | 32 | 0 | 3 | 1 | 83 | 0 | 4 | 45 | 255 | <10 | 0.53 | 113.7 | <0.01 | 0 | 2 | 0 |

| Grades: Grade 0=0, Grade 1=1, Grade 2=2, Grade 3=3 |
| --- |
| Indications: intolerance=1, preference=2, mass compression=3, Failure of I-131=4 |
| SETA: selective embolization of thyroid artery, 0=no , 1=yes |
| RFA: radiofrequency ablation, 0=no, 1=yes |
| Resection: TT, total thyroidectomy=1; NT, near-total thyroidectomy=2; ST-D, subtotal-Downhill=3; ST-BL, subtotal bilateral=4 |
| I-PTH: intact parathyroid hormone |
| Drainage 0=no,1=yes |
